# Supplementary material for: Genomic Determinants of Lethality and Therapeutic Vulnerability in Castration-Resistant Prostate Cancer
Source: bioRxiv. 2026 Jun 20:2026.06.18.733209. Preprint. [Version 1] doi: 10.64898/2026.06.18.733209 (PMC13411107; doi:10.64898/2026.06.18.733209)
Supplement: Supplementary file 1 [file media-1.pdf]

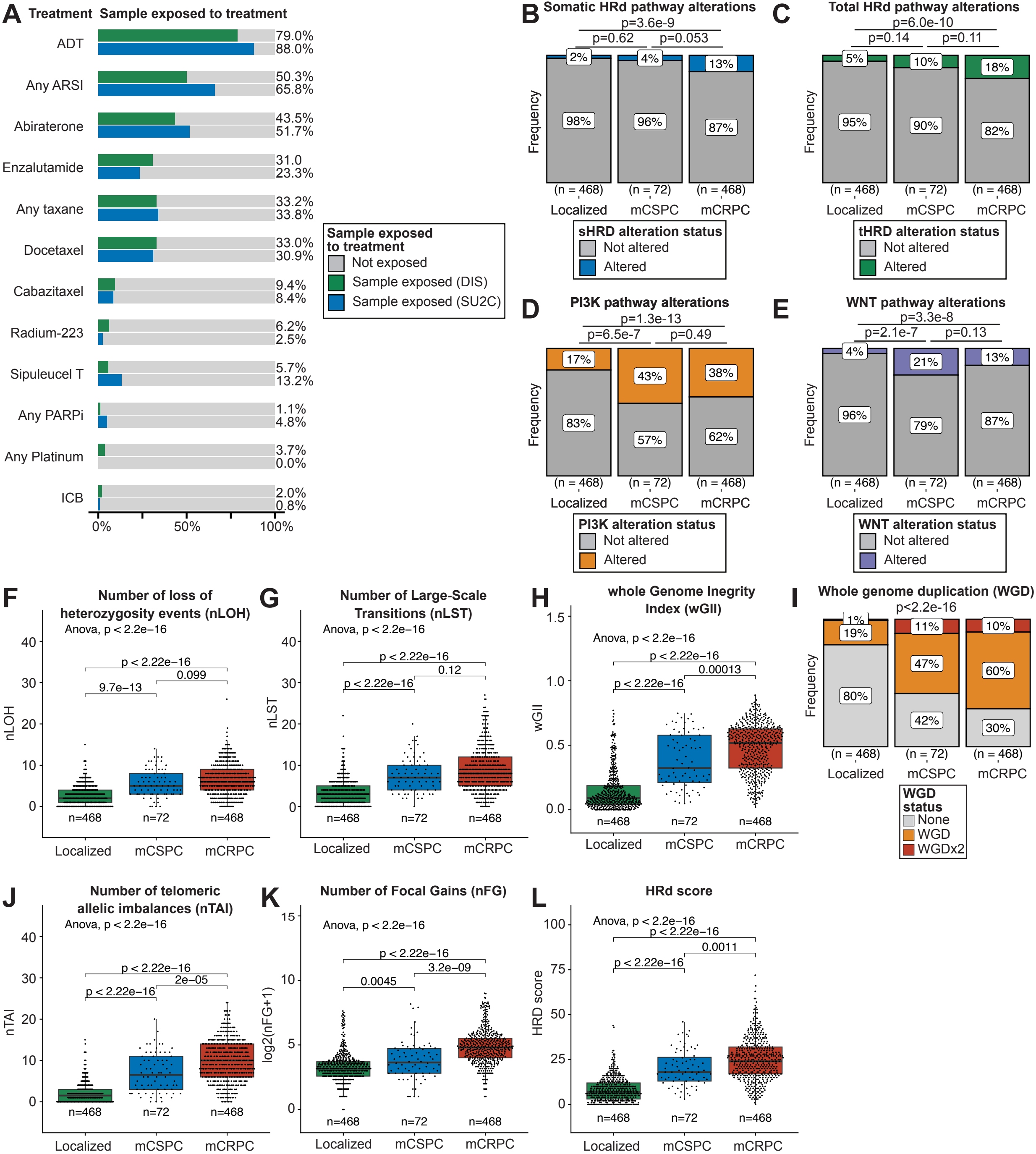

A Treatment Sample exposed to treatment
ADT 79.0% 88.0%
Any ARSI 50.3% 65.8%
Abiraterone 43.5% 51.7%
Enzalutamide 31.0 23.3%
Any taxane 33.2% 33.8%
Docetaxel 33.0% 30.9%
Cabazitaxel 9.4% 8.4%
Radium-223 6.2% 2.5%
Sipuleucel T 5.7% 13.2%
Any PARPi 1.1% 4.8%
Any Platinum 3.7% 0.0%
ICB 2.0% 0.8%
0% 50% 100%
Sample exposed to treatment
Not exposed
Sample exposed (DIS)
Sample exposed (SU2C)
B Somatic HRd pathway alterations
p=3.6e-9
p=0.62 p=0.053
2% 4% 13%
98% 96% 87%
Frequency
(n = 468) (n = 72) (n = 468)
Localized mCSPC mCRPC
sHRD alteration status
Not altered
Altered
C Total HRd pathway alterations
p=6.0e-10
p=0.14 p=0.11
5% 10% 18%
95% 90% 82%
Frequency
(n = 468) (n = 72) (n = 468)
Localized mCSPC mCRPC
tHRD alteration status
Not altered
Altered
D PI3K pathway alterations
p=1.3e-13
p=6.5e-7 p=0.49
17% 43% 38%
83% 57% 62%
Frequency
(n = 468) (n = 72) (n = 468)
Localized mCSPC mCRPC
PI3K alteration status
Not altered
Altered
E WNT pathway alterations
p=3.3e-8
p=2.1e-7 p=0.13
4% 21% 13%
96% 79% 87%
Frequency
(n = 468) (n = 72) (n = 468)
Localized mCSPC mCRPC
WNT alteration status
Not altered
Altered
F Number of loss of heterozygosity events (nLOH)
Anova, p < 2.2e−16
p < 2.22e−16
0.099
9.7e−13
nLOH
n=468 n=72 n=468
Localized mCSPC mCRPC
G Number of Large-Scale Transitions (nLST)
Anova, p < 2.2e−16
p < 2.22e−16
0.12
p < 2.22e−16
nLST
n=468 n=72 n=468
Localized mCSPC mCRPC
H whole Genome Inegrity Index (wGII)
Anova, p < 2.2e−16
p < 2.22e−16
0.00013
p < 2.22e−16
wGII
n=468 n=72 n=468
Localized mCSPC mCRPC
I Whole genome duplication (WGD)
p<2.2e-16
1% 19% 80%
11% 47% 42%
10% 60% 30%
Frequency
(n = 468) (n = 72) (n = 468)
Localized mCSPC mCRPC
WGD status
None
WGD
WGDx2
J Number of telomeric allelic imbalances (nTAI)
Anova, p < 2.2e−16
p < 2.22e−16
2e−05
p < 2.22e−16
nTAI
n=468 n=72 n=468
Localized mCSPC mCRPC
K Number of Focal Gains (nFG)
Anova, p < 2.2e−16
p < 2.22e−16
3.2e−09
0.0045
log2(nFG+1)
n=468 n=72 n=468
Localized mCSPC mCRPC
L HRd score
Anova, p < 2.2e−16
p < 2.22e−16
0.0011
p < 2.22e−16
HRD score
n=468 n=72 n=468
Localized mCSPC mCRPC

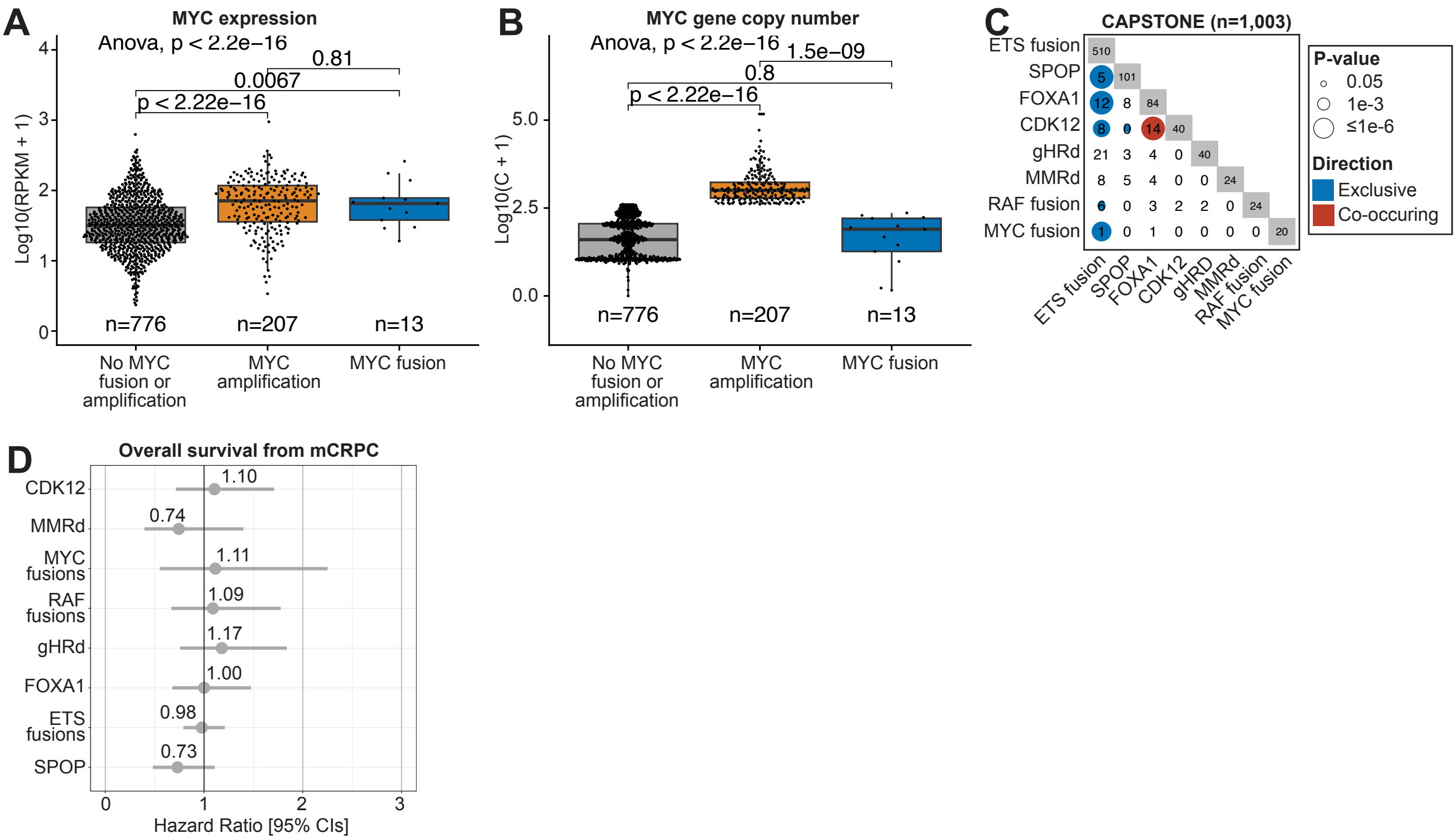

A
MYC expression
Anova, p < 2.2e−16
0.81
0.0067
p < 2.22e−16
Log10(RPKM + 1)
n=776
n=207
n=13
No MYC fusion or amplification
MYC amplification
MYC fusion
B
MYC gene copy number
Anova, p < 2.2e−16
1.5e−09
0.8
p < 2.22e−16
Log10(C + 1)
n=776
n=207
n=13
No MYC fusion or amplification
MYC amplification
MYC fusion
C
CAPSTONE (n=1,003)
ETS fusion
SPOP
FOXA1
CDK12
gHRd
MMRd
RAF fusion
MYC fusion
gHRD
P-value
0.05
1e-3
≤1e-6
Direction
Exclusive
Co-occuring
D
Overall survival from mCRPC
CDK12
MMRd
MYC fusions
RAF fusions
gHRd
FOXA1
ETS fusions
SPOP
1.10
0.74
1.11
1.09
1.17
1.00
0.98
0.73
Hazard Ratio [95% CIs]

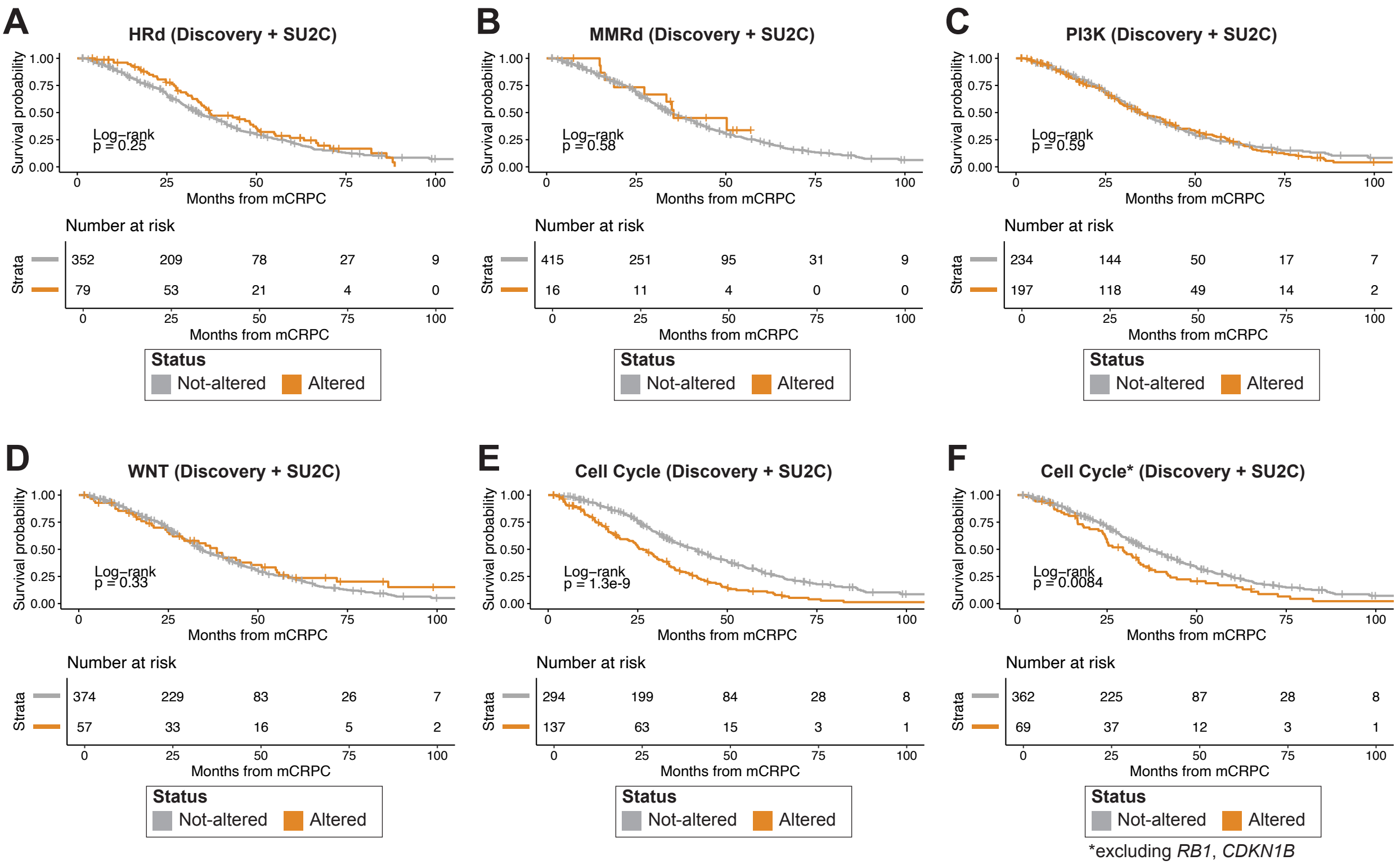
A
HRd (Discovery + SU2C)
Log−rank p = 0.25
B
MMRd (Discovery + SU2C)
Log−rank p = 0.58
C
PI3K (Discovery + SU2C)
Log−rank p = 0.59
D
WNT (Discovery + SU2C)
Log−rank p = 0.33
E
Cell Cycle (Discovery + SU2C)
Log−rank p = 1.3e-9
F
Cell Cycle* (Discovery + SU2C)
Log−rank p = 0.0084
Survival probability
Months from mCRPC
Number at risk
Strata
Status
Not-altered
Altered
*excluding RB1, CDKN1B

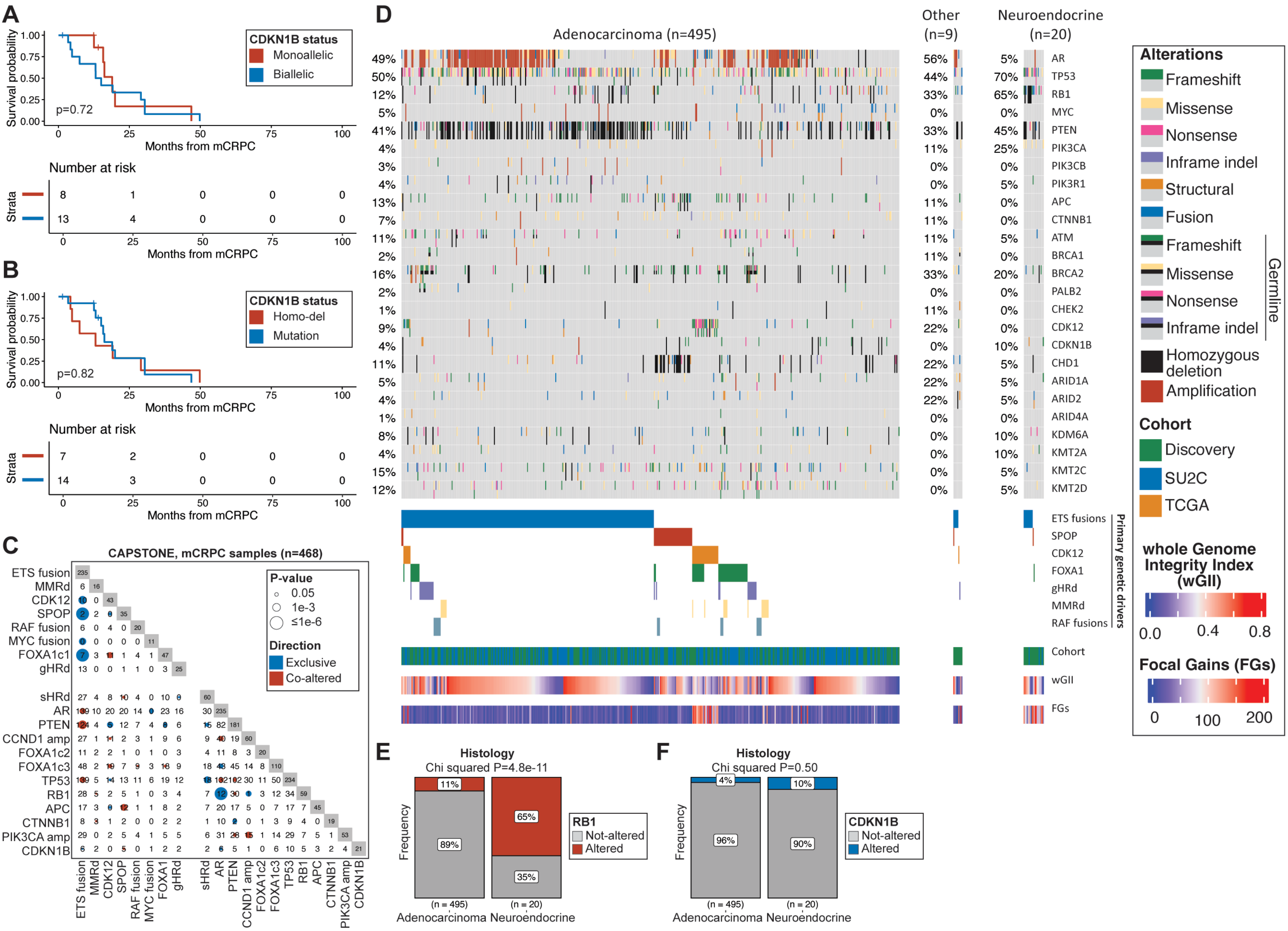
A
CDKN1B status
Monoallelic
Biallelic
Survival probability
p=0.72
Months from mCRPC
Number at risk
Strata
8 1 0 0 0
13 4 0 0 0
B
CDKN1B status
Homo-del
Mutation
Survival probability
p=0.82
Months from mCRPC
Number at risk
Strata
7 2 0 0 0
14 3 0 0 0
C
CAPSTONE, mCRPC samples (n=468)
P-value
0.05
1e-3
≤1e-6
Direction
Exclusive
Co-altered
ETS fusion
MMRd
CDK12
SPOP
RAF fusion
MYC fusion
FOXA1c1
gHRd
sHRd
AR
PTEN
CCND1 amp
FOXA1c2
FOXA1c3
TP53
RB1
APC
CTNNB1
PIK3CA amp
CDKN1B
D
Adenocarcinoma (n=495)
Other (n=9)
Neuroendocrine (n=20)
AR
TP53
RB1
MYC
PTEN
PIK3CA
PIK3CB
PIK3R1
APC
CTNNB1
ATM
BRCA1
BRCA2
PALB2
CHEK2
CDK12
CDKN1B
CHD1
ARID1A
ARID2
ARID4A
KDM6A
KMT2A
KMT2C
KMT2D
ETS fusions
SPOP
CDK12
FOXA1
gHRd
MMRd
RAF fusions
Primary genetic drivers
Cohort
wGII
FGs
Alterations
Frameshift
Missense
Nonsense
Inframe indel
Structural
Fusion
Frameshift
Missense
Nonsense
Inframe indel
Germline
Homozygous deletion
Amplification
Cohort
Discovery
SU2C
TCGA
whole Genome Integrity Index (wGII)
0.0 0.4 0.8
Focal Gains (FGs)
0 100 200
E
Histology
Chi squared P=4.8e-11
Frequency
11%
89%
65%
35%
(n = 495)
(n = 20)
Adenocarcinoma
Neuroendocrine
RB1
Not-altered
Altered
F
Histology
Chi squared P=0.50
Frequency
4%
96%
10%
90%
(n = 495)
(n = 20)
Adenocarcinoma
Neuroendocrine
CDKN1B
Not-altered
Altered

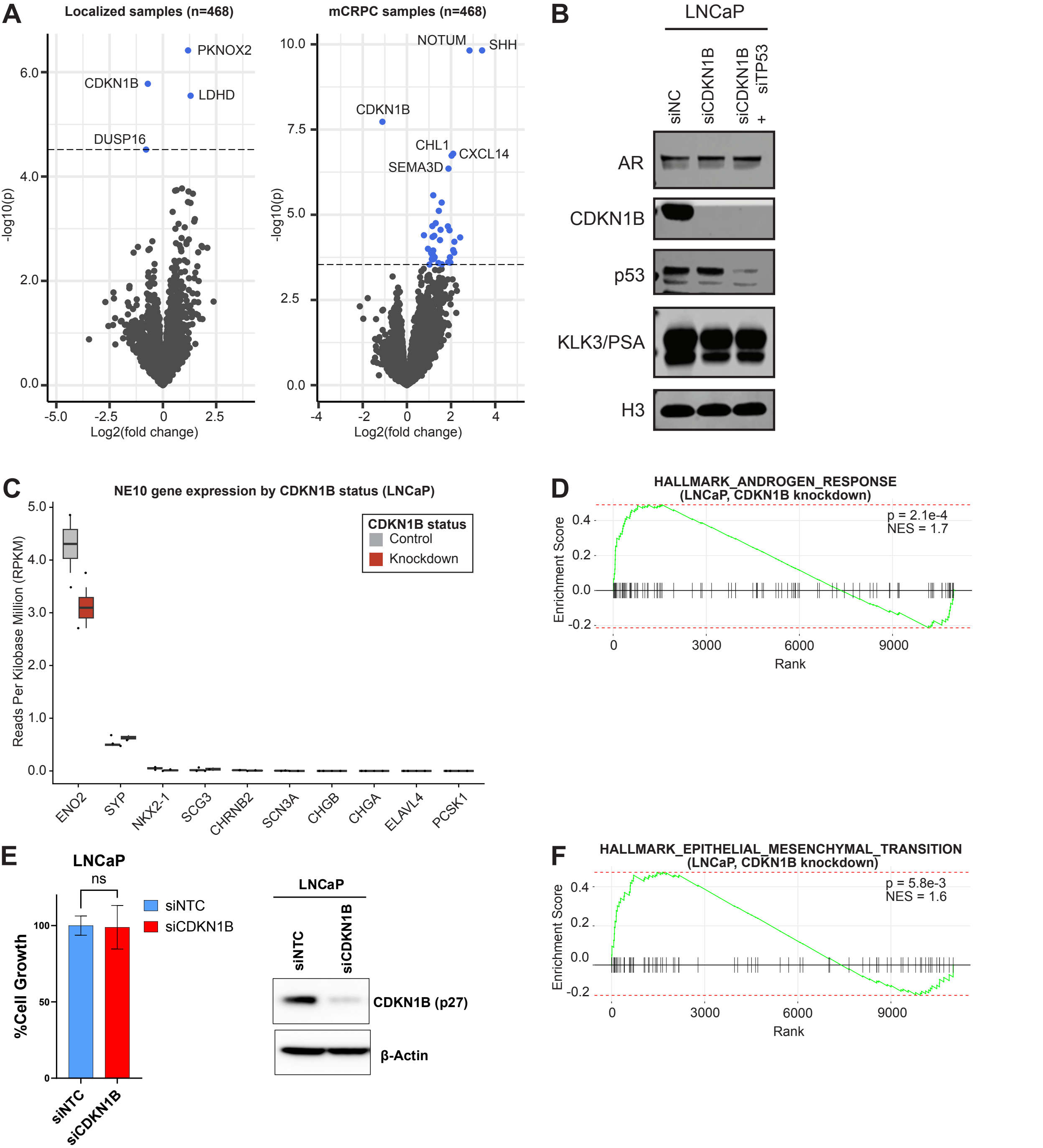
A
Localized samples (n=468)
PKNOX2
CDKN1B
LDHD
DUSP16
-log10(p)
Log2(fold change)
mCRPC samples (n=468)
NOTUM
SHH
CDKN1B
CHL1
CXCL14
SEMA3D
B
LNCaP
siNC
siCDKN1B
siCDKN1B + siTP53
AR
CDKN1B
p53
KLK3/PSA
H3
C
NE10 gene expression by CDKN1B status (LNCaP)
CDKN1B status
Control
Knockdown
Reads Per Kilobase Million (RPKM)
ENO2
SYP
NKX2-1
SCG3
CHRNB2
SCN3A
CHGB
CHGA
ELAVL4
PCSK1
D
HALLMARK_ANDROGEN_RESPONSE
(LNCaP, CDKN1B knockdown)
p = 2.1e-4
NES = 1.7
Enrichment Score
Rank
E
LNCaP
ns
siNTC
siCDKN1B
%Cell Growth
LNCaP
siNTC
siCDKN1B
CDKN1B (p27)
β-Actin
F
HALLMARK_EPITHELIAL_MESENCHYMAL_TRANSITION
(LNCaP, CDKN1B knockdown)
p = 5.8e-3
NES = 1.6
Enrichment Score
Rank

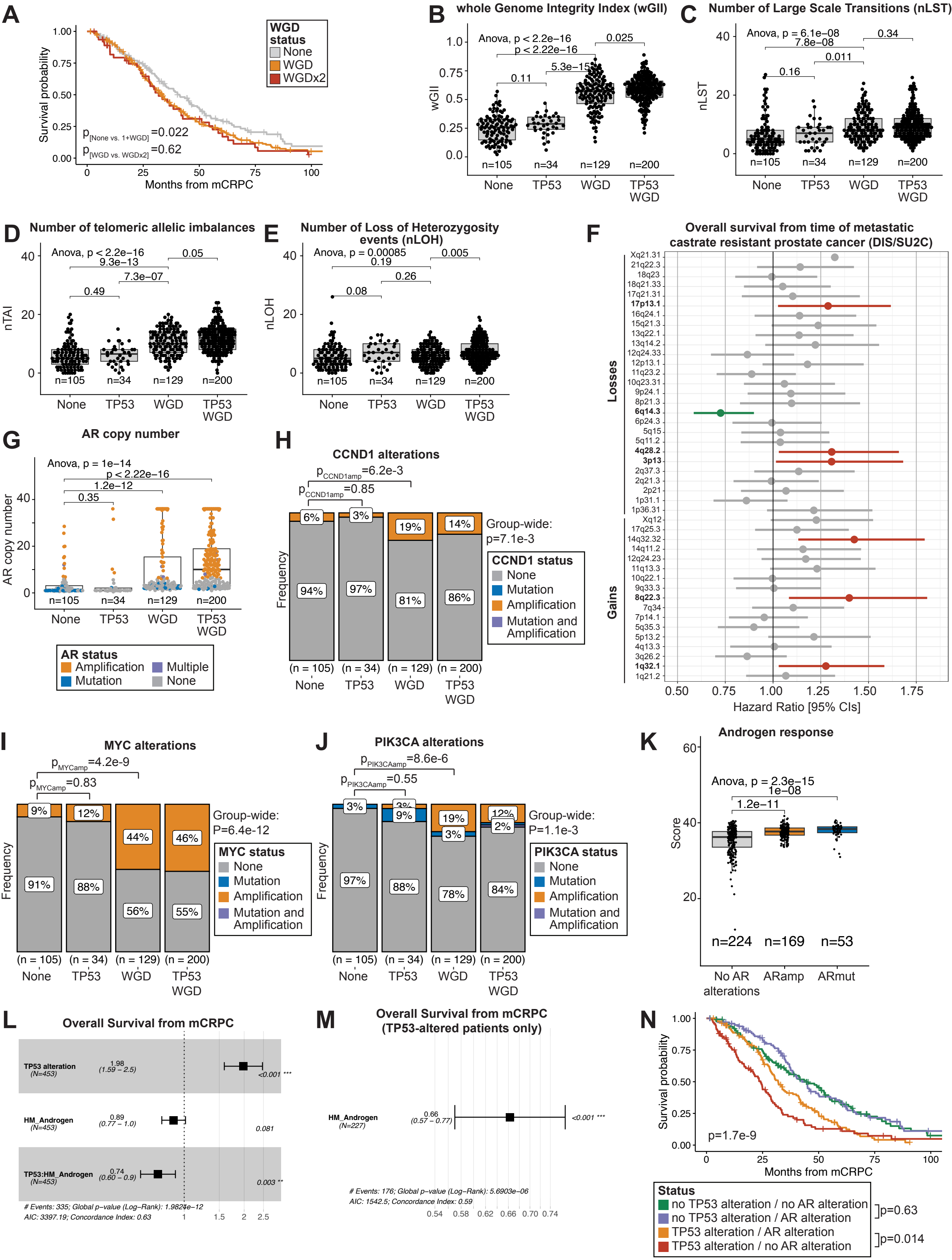

A
WGD status
None
WGD
WGDx2
Survival probability
Months from mCRPC
p[None vs. 1+WGD] =0.022
p[WGD vs. WGDx2] =0.62
B
whole Genome Integrity Index (wGII)
wGII
Anova, p < 2.2e−16
p < 2.22e−16
0.025
5.3e−15
0.11
n=105 n=34 n=129 n=200
None TP53 WGD TP53 WGD
C
Number of Large Scale Transitions (nLST)
nLST
Anova, p = 6.1e−08
7.8e−08
0.34
0.011
0.16
n=105 n=34 n=129 n=200
None TP53 WGD TP53 WGD
D
Number of telomeric allelic imbalances
nTAI
Anova, p < 2.2e−16
9.3e−13
0.05
7.3e−07
0.49
n=105 n=34 n=129 n=200
None TP53 WGD TP53 WGD
E
Number of Loss of Heterozygosity events (nLOH)
nLOH
Anova, p = 0.00085
0.19
0.005
0.26
0.08
n=105 n=34 n=129 n=200
None TP53 WGD TP53 WGD
F
Overall survival from time of metastatic castrate resistant prostate cancer (DIS/SU2C)
Losses
Xq21.31 21q22.3 18q23 18q21.33 17q21.31 17p13.1 16q24.1 15q21.3 13q22.1 13q14.2 12q24.33 12p13.1 11q23.2 10q23.31 9p24.1 8p21.3 6q14.3 6p24.3 5q15 5q11.2 4q28.2 3p13 2q37.3 2q21.3 2p21 1p31.1 1p36.31
Gains
Xq12 17q25.3 14q32.32 14q11.2 12q24.23 11q13.3 10q22.1 9q33.3 8q22.3 7q34 7p14.1 5q35.3 5p13.2 4q13.3 3q26.2 1q32.1 1q21.2
Hazard Ratio [95% CIs]
G
AR copy number
Anova, p = 1e−14
p < 2.22e−16
1.2e−12
0.35
n=105 n=34 n=129 n=200
None TP53 WGD TP53 WGD
AR status
Amplification
Multiple
Mutation
None
H
CCND1 alterations
pCCND1amp=6.2e-3
pCCND1amp=0.85
Frequency
6% 3% 19% 14%
94% 97% 81% 86%
Group-wide: p=7.1e-3
CCND1 status
None
Mutation
Amplification
Mutation and Amplification
(n = 105) (n = 34) (n = 129) (n = 200)
None TP53 WGD TP53 WGD
I
MYC alterations
pMYCamp=4.2e-9
pMYCamp=0.83
9% 12% 44% 46%
91% 88% 56% 55%
Group-wide: P=6.4e-12
MYC status
None
Mutation
Amplification
Mutation and Amplification
J
PIK3CA alterations
pPIK3CAamp=8.6e-6
pPIK3CAamp=0.55
3% 9% 19% 3% 12% 2%
97% 88% 78% 84%
Group-wide: P=1.1e-3
PIK3CA status
None
Mutation
Amplification
Mutation and Amplification
K
Androgen response
Score
Anova, p = 2.3e−15
1e−08
1.2e−11
n=224 n=169 n=53
No AR alterations
ARamp
ARmut
L
Overall Survival from mCRPC
TP53 alteration (N=453) 1.98 (1.59 – 2.5) <0.001 ***
HM_Androgen (N=453) 0.89 (0.77 – 1.0) 0.081
TP53:HM_Androgen (N=453) 0.74 (0.60 – 0.9) 0.003 **
Events: 335; Global p-value (Log-Rank): 1.9824e−12
AIC: 3397.19; Concordance Index: 0.63
M
Overall Survival from mCRPC (TP53-altered patients only)
HM_Androgen (N=227) 0.66 (0.57 – 0.77) <0.001 ***
Events: 176; Global p-value (Log-Rank): 5.6903e−06
AIC: 1542.5; Concordance Index: 0.59
N
p=1.7e-9
Status
no TP53 alteration / no AR alteration
no TP53 alteration / AR alteration
TP53 alteration / AR alteration
TP53 alteration / no AR alteration
p=0.63
p=0.014

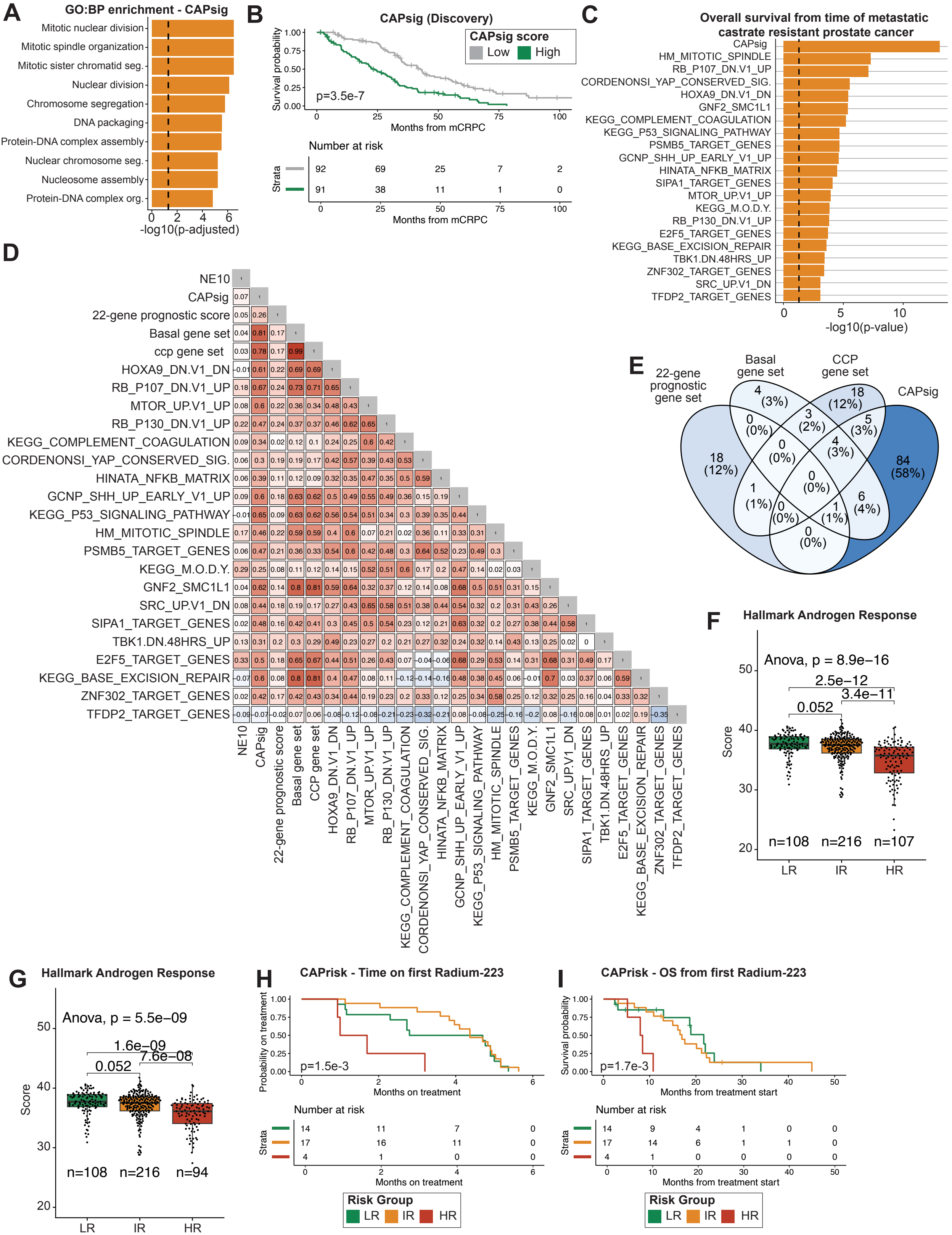

A
GO:BP enrichment - CAPsig
Mitotic nuclear division
Mitotic spindle organization
Mitotic sister chromatid seg.
Nuclear division
Chromosome segregation
DNA packaging
Protein-DNA complex assembly
Nuclear chromosome seg.
Nucleosome assembly
Protein-DNA complex org.
-log10(p-adjusted)
B
CAPsig (Discovery)
CAPsig score
Low
High
Survival probability
p=3.5e-7
Months from mCRPC
Number at risk
Strata
92 69 25 7 2
91 38 11 1 0
C
Overall survival from time of metastatic castrate resistant prostate cancer
CAPsig
HM_MITOTIC_SPINDLE
RB_P107_DN.V1_UP
CORDENONSI_YAP_CONSERVED_SIG.
HOXA9_DN.V1_DN
GNF2_SMC1L1
KEGG_COMPLEMENT_COAGULATION
KEGG_P53_SIGNALING_PATHWAY
PSMB5_TARGET_GENES
GCNP_SHH_UP_EARLY_V1_UP
HINATA_NFKB_MATRIX
SIPA1_TARGET_GENES
MTOR_UP.V1_UP
KEGG_M.O.D.Y.
RB_P130_DN.V1_UP
E2F5_TARGET_GENES
KEGG_BASE_EXCISION_REPAIR
TBK1.DN.48HRS_UP
ZNF302_TARGET_GENES
SRC_UP.V1_DN
TFDP2_TARGET_GENES
-log10(p-value)
D
NE10
CAPsig
22-gene prognostic score
Basal gene set
ccp gene set
E
22-gene prognostic gene set
Basal gene set
CCP gene set
CAPsig
84 (58%)
F
Hallmark Androgen Response
Anova, p = 8.9e−16
2.5e−12
3.4e−11
0.052
Score
n=108 n=216 n=107
LR IR HR
G
Hallmark Androgen Response
Anova, p = 5.5e−09
1.6e−09
7.6e−08
0.052
Score
n=108 n=216 n=94
LR IR HR
H
CAPrisk - Time on first Radium-223
Probability on treatment
p=1.5e-3
Months on treatment
Number at risk
Strata
14 11 7 0
17 16 11 0
4 1 0 0
Risk Group
LR IR HR
I
CAPrisk - OS from first Radium-223
Survival probability
p=1.7e-3
Months from treatment start
Number at risk
Strata
14 9 4 1 0 0
17 14 6 1 1 0
4 1 0 0 0 0
Risk Group
LR IR HR
